# Supplementary material for: DNA-Templated Silver Nanoclusters as Dual-Mode Sensitive Probes for Self-Powered Biosensor Fueled by Glucose
Source: Nanomaterials (Basel). 2023 Apr 7;13(8):1299. doi: 10.3390/nano13081299 (PMC10145323; doi:10.3390/nano13081299)
Supplement: Supplementary file 1 [file nanomaterials-13-01299-s001.zip › nanomaterials-2321596-supplementary.pdf]

Supplementary Materials

DNA–Templated Silver Nanoclusters as Dual-Mode Sensitive Probes for Self-Powered Biosensor Fueled by Glucose.

Akhilesh Kumar Gupta[1], and Alexey V. Krasnoslobodtsev[1*]

[1] Department of Physics, University of Nebraska Omaha, Omaha, NE 68182, USA.

***** Correspondence: akrasnos@unomaha.edu; Tel.: +1-402-554-3723.

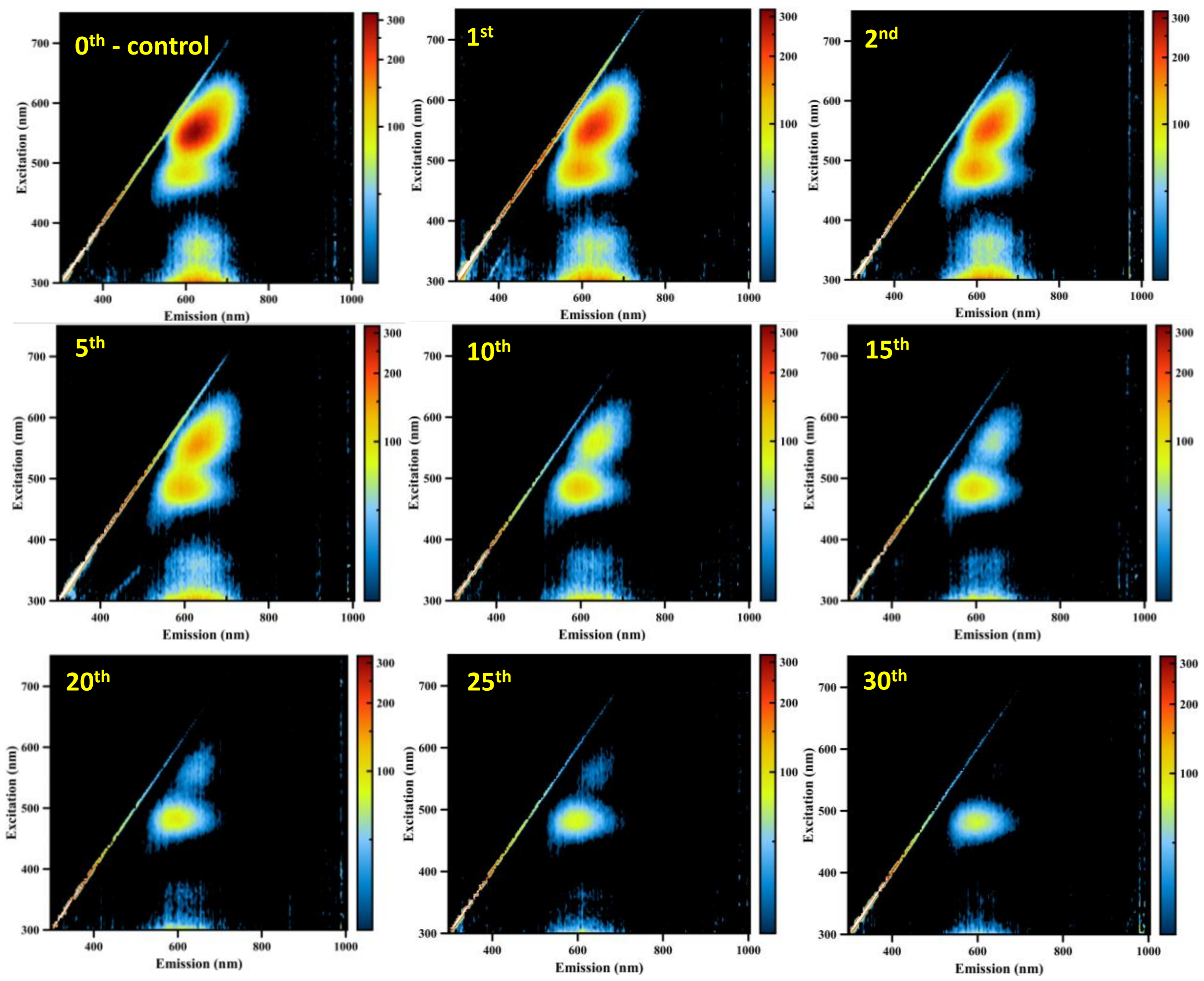

Figure S1. Representative EEMS patterns for probing performance of the optical readout for the mixture of 100 µL of 10 µM AgNCs@DNA, 5 µL of 1 mM GOx with the successive addition of 5 µL (500µM glucose) solution. Numbers indicate the nth addition of the 5 µL glucose solution. The EEM patterns show gradual decline of the "red" emissive peak with increased glucose concentration which was then used for evaluation of the sensor's optical readout strategy.

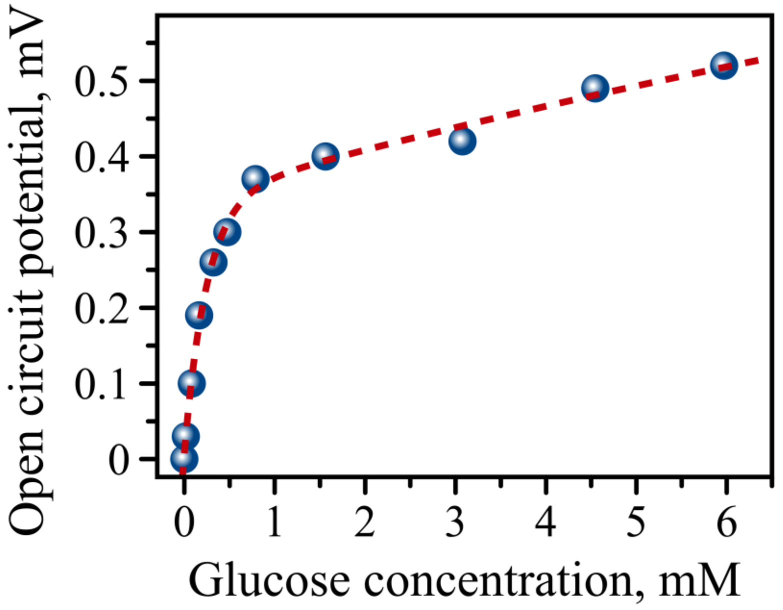

Figure S2. Open circuit potential (OCP) dependence on glucose concentration. The dependence indicates that the BFC performance is still controlled by the kinetics of the enzymatic reaction, GOx + glucose, but the potential increases even at high glucose concentrations providing the possibility for power output and the design's operation as BFC.

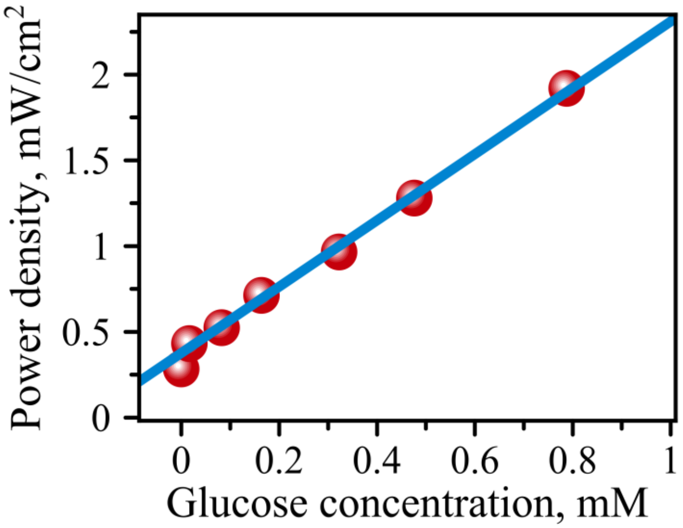

Figure S3. Enlarged section of the power density vs $C_{glucose}$ plot in the range of concentrations between 0 and 1 mM (highlighted in blue in **Figure 3C**). Solid blue line is the linear approximation, a*x+b to the data points (a= $1.98*10^{-6}$, b=$3.5*10^{-4}$) which was used to estimate LOD level yielding 29.2 μM of glucose concentration.
